# Clinical outcomes of weekly adalimumab in refractory non-infectious paediatric uveitis and the role of serum drug levels and anti-adalimumab antibodies

**DOI:** 10.1038/s41433-026-04460-x

**Published:** 2026-05-29

**Authors:** Amelia Rees, Raheej Khan, Jessy Choi, Clare Nash, Daniel Hawley, Sasa Pockar, Shiao Wei Wong, Guilia Varnier, Vinod Sharma, Alice Chieng, Jane Ashworth

**Affiliations:** 1 St George’s University NHS Foundation Trust, London, UK; 2 Manchester Royal Eye Hospital, Manchester, UK; 3 Sheffield Children’s Hospital, Sheffield, UK; 4 Royal Manchester Children’s Hospital, Manchester, UK; 5 The University of Manchester, Manchester, UK

**Keywords:** Medical research, Paediatrics, Predictive markers, Uveal diseases

## Abstract

**Background/objectives:**

Adalimumab (ADA) is a well-established treatment for refractory paediatric uveitis; however, some patients remain inadequately controlled on standard biweekly dosing. Weekly ADA injections are increasingly used, though evidence in paediatric cohorts remains limited. This study evaluates outcomes of escalation to weekly ADA in children with refractory uveitis and explores the relevance of serum drug levels and anti-ADA antibodies (AAA).

**Subjects/methods:**

Patients with idiopathic uveitis at two tertiary centres in the UK treated with weekly adalimumab were retrospectively reviewed. Demographic, clinical and laboratory data were collected, including age at escalation, systemic diagnosis, serum ADA levels, AAA titres pre- and post-escalation, clinical response and complications. Treatment success was defined as ≤0.5+ anterior chamber cells and <2 drops/day of steroid eye drops at 3 months, or most recent follow-up where escalation occurred within 3 months of review.

**Results:**

15 patients were included. Mean age at escalation was 11 years (range: 3-17). 11 (73%) had a systemic diagnosis; most commonly juvenile idiopathic arthritis (60%). Median serum level pre-escalation was 10.1 mg/L (IQR: 7.8–11.9) and 18.1 mg/L (IQR: 14.8–22.1) post escalation. AAAs were detectable in 7/15 (47%) pre-escalation, 2/15 (13.3%) post-escalation. Success occurred in 10/15 (67%), with visual acuity stable and improved in 93%. No serious systemic adverse events were reported.

**Conclusions:**

Weekly ADA offers a safe and effective treatment alternative for children with refractory uveitis, including those with AAAs. These findings support its inclusion in future treatment pathways, though the predictive value of serum levels and AAAs remains uncertain and warrants further investigation.

## Introduction

Paediatric chronic non-infectious anterior uveitis (pCAU) is a severe ocular inflammatory disease that can result in significant ocular comorbidities, including cataracts, glaucoma, band keratopathy, retinal detachment, macular and optic disc oedema, leading to permanent structural damage and irreversible vision loss [1–3]. The most well-recognised systemic association of pCAU is juvenile idiopathic arthritis (JIA), the most frequently occurring paediatric rheumatic disease, with up to 38% of patients reported to develop ocular involvement [2, 4–7]. The standard management of pCAU refractory to topical therapies involves systemic immunosuppressive therapy, including disease-modifying anti-rheumatic drugs (DMARDs) [8]. In November 2015 the National Health Service (NHS) England approved the use of adalimumab (ADA) for the treatment of sight threatening severe uveitis in children and young people, when other treatments have been ineffective. ADA has since revolutionised treatment outcomes [9–12].

ADA is a subcutaneously administered, fully-humanised recombinant monoclonal antibody which targets and blocks tumour necrosis factor alpha (TNF-α). ADA is currently the only biologic agent approved for use in the management of non-infectious uveitis in both adult and paediatric cohorts [9, 13] with the exception of infliximab which is approved for JIA, and by extension, JIA-related uveitis [14]. ADA has been shown to be safe and effective in controlling intraocular inflammation, reducing frequency of uveitic flares and reducing reliance on topical corticosteroids [1–3, 9, 10, 13, 15–18]. The approved dosing schedule for ADA is weight-dependent, with biweekly administrations of 40 mg for children weighing ≥30 kg, and 20 mg for those weighing <30 kg [10]. While standard biweekly dosing is effective for most patients at controlling ocular inflammation, a subset of patients show persistent disease [10]. Weekly ADA is approved within the NHS for the treatment of refractory rheumatoid arthritis, inflammatory bowel disease [19, 20] and hidradenitis suppurativa [21–23]. Weekly dosing is being increasingly considered in refractory uveitis, with emerging evidence supporting its safety and efficacy as an escalation strategy for refractory CAU in both adults [13, 24] and children [1, 10, 16]. Evidence supporting this approach in non-uveitic JIA is scarce, likely due to the availability of alternative treatment options reducing the need to explore weekly ADA in this population. However, the few available reports suggest encouraging outcomes for refractory patients [16, 25].

Although some studies have supported the use of weekly ADA injections, this remains an off-label approach, and evidence specific to paediatric uveitis is limited [1, 10, 16]. These are retrospective observational studies, thus constituting level 3-4 evidence. This study aims to evaluate clinical outcomes following escalation to weekly ADA in children with refractory pCAU, and to explore the role of serum drug levels and anti-adalimumab antibody titres in guiding treatment decisions.

## Methods

This retrospective observational study was conducted across two tertiary specialist paediatric uveitis centres in the United Kingdom: Manchester Royal Eye Hospital and Sheffield Children’s Hospital. Electronic medical records were reviewed in detail for all paediatric patients who underwent escalation from standard biweekly to weekly ADA dosing for pCAU between January 2024 and April 2025. This project was deemed service-evaluation work at both institutions involved, therefore, formal ethics approval was not required.

Inclusion criteria consisted of (1) a confirmed diagnosis of pCAU and (2) a documented escalation to weekly ADA due to inadequate disease control on biweekly dosing. Patients were excluded in cases of non-compliance with treatment (e.g. missed injections or deviation from prescribed regimen), or lack of follow-up.

Data collected included demographic information (date of birth, age at escalation, sex), systemic diagnosis, duration of disease and uveitis laterality. Treatment history was documented, including previous and concurrent systemic immunosuppressive therapies, topical corticosteroid use, and duration on biweekly ADA prior to escalation. Laboratory results included random serum ADA levels and anti-adalimumab antibody titres, where available, with measurements recorded before and after escalation.

The primary outcome was clinical response, with success defined as ≤0.5+ anterior chamber cells, < 2 drops/day of steroid eye drops and no concurrent systemic steroid use at 3 months following escalation, or at most recent review if escalation had occurred within 3 months. Secondary outcomes included presence or development of anti-drug antibodies and the documentation of any adverse events during the follow-up period. Statistical analysis was performed using Python (version 3.11) [26].

## Results

### Patient characteristics

All patients identified to have been escalated to weekly ADA were included, resulting in a total of 15 paediatric patients (6/15 female (40%), 9/15 male (60%), with a mean age at escalation to weekly ADA of 11 years (range: 3–17 years). All patients had a diagnosis of non-infectious pCAU; 11/15 (73%) had anterior uveitis, 1/15 (7%) had intermediate uveitis and 3/15 (20%) had panuveitis. The majority (13/15, 87%) were bilateral, with a minority unilateral (2/15, 13%). 11/15 (73%) had a concurrent systemic inflammatory condition: 9/15 (60%) had JIA-associated uveitis, while 1/15 (7%) was diagnosed with biopsy-confirmed tubulointerstitial nephritis and uveitis (TINU) syndrome and 1/15 (7%) with Blau syndrome. The remaining 4/15 (27%) had isolated ocular disease (idiopathic non-infectious uveitis). A summary of the baseline characteristics is demonstrated in Table 1.

**Table 1:** Baseline demographic and clinical characteristics of the 15 children with non-infectious uveitis at baseline, including age, sex, uveitis subtype, laterality and associated systemic conditions.

Characteristic		Value *n*(%)
**No. Patients (n)**		*n* = 15
**Age (Years)**		Mean: 11
		Range: 4-17
**Sex**		Male: 9 (60.0%)
		Female: 6 (40.0%)
**Uveitis Subtype**	**Anterior Uveitis**	11 (73.3%)
	**Intermediate Uveitis**	1 (6.7%)
	**Panuveitis**	3 (20.0%)
**Laterality**	**Unilateral**	2 (13%)
	**Bilateral**	13 (87%)
**Associated Systemic Condition**	**JIA**	9 (60%)
	**TINU**	1 (7%)
	**Sarcoid/Blau**	1 (7%)
	**None**	4 (27%)

All patients demonstrated persistent ocular inflammation despite consistent compliance with standard biweekly ADA and concurrent immunosuppression with therapeutic dosing of DMARDs; either methotrexate (MTX, 9/15, 60%) or mycophenolate mofetil (MMF, 6/15, 40%). Prior to escalation, 6/15 (40%) patients were on 20 mg biweekly and 9/15 (60%) on 40 mg biweekly. Mean duration on biweekly ADA prior to escalation was 1404 days (range: 202–4986 days); indicating that standard dosing was able to maintain disease control for a prolonged period in many patients before subsequent loss of efficacy necessitated escalation. ADA dosing remained weight appropriate throughout the study period.

### Treatment outcomes

At 3 months post-escalation, 10/15 (66.7%) achieved disease control. Of the remaining 5/15 (33%) patients who didn’t meet the treatment success criteria, 1/5 (20%, P3) had moderate residual inflammation (2+ anterior chamber [AC] cells) and did not meet corticosteroid criteria for treatment success. 1/5 (20%; P15) had mild residual inflammation (1 + AC cells) with ongoing frequent corticosteroid use and discontinued treatment after 1 month due to poor compliance. The remaining 3/5 (60%; P4, P8, P10) had quiescent inflammation ( < 0.5 + AC cells) but did not meet the corticosteroid criterion.

At baseline, AC inflammation ranged from 0 to 2+ cells. The majority of patients (12/15, 80%) demonstrated at least 1+ inflammation in the worse eye (Supplementary Table 1). Two patients (P6 and P9) with minimal AC inflammation at baseline (0 and 0.5 + , respectively) were escalated due to cystoid macular oedema (CMO) rather than AC activity alone.

Following escalation, 12/15 (80%) patients demonstrated an improvement in AC inflammation by at least one SUN grade. 7/15 (47%) improved by at least one full grade, including 5/15 (33%) who improved by two grades. 3/15 (20%) demonstrated no change in AC inflammation grade; one of these (P6) had no inflammation at baseline and no further CMO flares were observed during follow-up. The remaining two showed persistent inflammation and were classed at treatment failure at 3 months. Importantly, no patient experienced worsening of inflammation at 3 months.

At the time of writing, the mean duration of weekly ADA treatment was 230 days (range: 56-501 days).

### Biomarker analysis

#### Pre-escalation

**Fig. 1:**
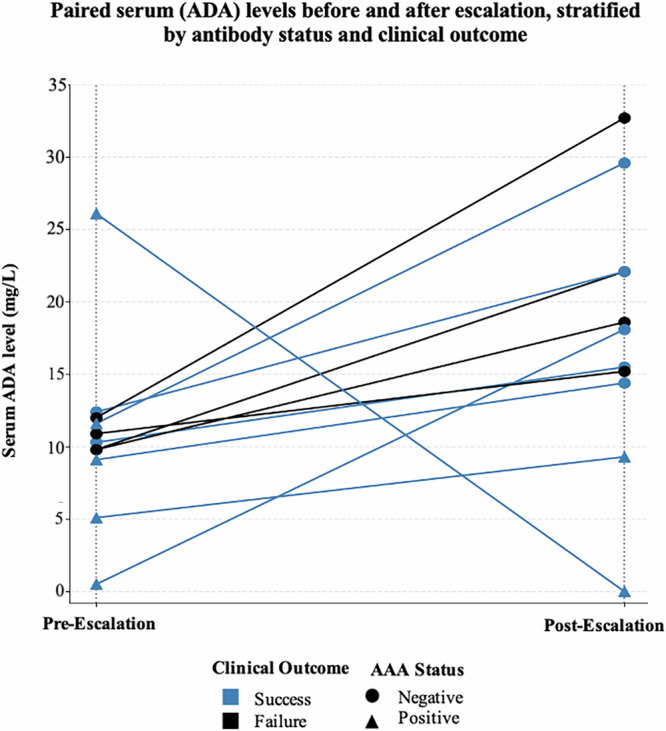
Paired random serum adalimumab (ADA) levels before and after escalation to weekly dosing, stratified by antibody status and clinical outcome. Each line represents an individual patient with complete pre- and post-escalation random serum ADA measurements (*n* = 11). Patients with missing data were excluded from the figure. Lines are coloured by clinical outcome at follow-up (blue = treatment success; black = treatment failure). Marker shape denotes anti-adalimumab antibody (AAA) status at the corresponding timepoint (circle = AAA-negative; triangle = AAA-positive). Treatment success was defined according to pre-specified disease control criteria. Points are aligned exactly at “Pre-escalation” and “Post-escalation” timepoints.

Table 2 summarises paired serum ADA level and anti-adalimumab and treatment outcomes before and after escalation to weekly dosing; these results are represented in Fig. 1. Pre-escalation serum ADA levels were available for 14/15 (93%) patients, ranging from 0.5–26.1 mg/L (median 10.1 mg/L, IQR: 7.8–11.9 mg/L). When stratified by outcome, pre-escalation ADA levels were similar between responders (range 0.5–26.1 mg/L, median 10.3 mg/L, IQR: 7.4–12.4 mg/L) and non-responders (range 5.8–12.0 mg/L, median 9.8 mg/L, IQR: 9.8–10.9 mg/L). The drug-level difference between the success and failure groups was not statistically significant (Mann-Whitney U = 24.0, *p* = 0.89).

**Table 2:** Paired random serum adalimumab levels, anti-adalimumab antibody status, and treatment outcomes before and after escalation from biweekly to weekly dosing in 15 children with refractory non-infectious uveitis.

*PATIENT*	*BIWEEKLY ADALIMUMAB*			*WEEKLY ADALIMUMAB*			*TREATMENT OUTCOME*
	DOSE	DRUG LEVEL	ANTIBODY STATUS	DOSE	DRUG LEVEL	ANTIBODY STATUS	
*1*	40 mg	10.3	Negative	40 mg	15.5	Negative	Success
*2*	20 mg	12.9	Negative	40 mg	Not recorded	Not recorded	Success
*3*	20 mg	9.8	Negative	40 mg	18.6	Negative	Failure
*4*	40 mg	9.8	Negative	40 mg	22.1	Negative	Failure
*5*	40 mg	12.4	Negative	40 mg	22.1	Negative	Success
*6*	20 mg	7.4	Negative	20 mg	Not recorded	Negative	Success
*7*	40 mg	<0.5	Positive (195 AU/mL)	40 mg	18.1	Negative	Success
*8*	40 mg	10.9	Negative	40 mg	15.2	Negative	Failure
*9*	40 mg	9.1	Positive (29.7 AU/mL)	40 mg	14.4	Negative	Success
*10*	20 mg	12	Negative	20 mg	32.7	Negative	Failure
*11*	20 mg	11.6	Positive (71 AU/mL)	40 mg	29.6	Negative	Success
*12*	20 mg	5.1	Positive (41 AU/mL)	20 mg	9.3	Positive (959 AU/mL)	Success
*13*	40 mg	26.1	Positive (12 AU/mL)	40 mg	0	Positive (12 AU/mL)	Success
*14*	40 mg	Not recorded	Positive (16 AU/mL)	40 mg	Not recorded	Not recorded	Success
*15*	40 mg	5.8	Positive (79 AU/mL)	40 mg	Not recorded	Negative	Failure

Among the baseline AAA negative patients (8/15, 53%), random serum levels ranged from 7.4–12.9 mg/L (median 10.6 mg/L, IQR: 9.8–12.1 mg/L), with 4/8 (50%) achieving treatment success. Of the baseline AAA-positive patients (7/15, 47%), serum ADA levels were available for 6/7 (86%) patients and ranged from 0.5–26.1 mg/L (median 7.5 mg/L, IQR: 5.3–11.0 mg/L) and 6/7 (86%) achieved success. The antibody titres in the seropositive patients ranged from 12-195 AU/mL (median 41 AU/mL, IQR: 16-71 AU/mL) with higher antibody generally associated with lower serum ADA levels (Spearman’s ρ = –0.71, *p* = 0.11), although this did not reach statistical significance, likely reflecting the small sample size and random serum sampled opportunistically when the child attended clinic. Fisher’s exact test demonstrated no significant association between pre-escalation antibody status and treatment outcome (*p* = 0.28). The odds of treatment success were numerically higher in AAA-positive patients (odds ratio = 6), although this did not reach statistical significance.

#### Post-escalation

Post-escalation, random serum ADA levels were available for 11/15 (73%) patients, ranging from 0.0–32.7 mg/L (median 18.1 mg/L, IQR: 14.8–22.1 mg/L) with the majority demonstrating marked rises in drug level. When divided by treatment outcome, ADA random drug levels were higher in the failure group (range 15.2–32.7 mg/L, median 20.4 mg/L, IQR 16.9–27.4 mg/L) than the treatment success group (range 0.0–29.6 mg/L, median 15.5 mg/L, IQR 9.3–22.1 mg/L), though this difference was not statistically significant (Mann-Whitney U = 7.5, *p* = 0.32).

AAA status was available for 13/15 patients. Of the 7 AAA-positive patients at baseline, 5/7 (71%) had post escalation drug levels available (range 0.0–29.6 mg/L, median 14.4 mg/L, IQR 9.3–18.1). Of these 4/7 (57%) seroconverted to negative, 2/7 (29%) remained AAA-positive and 1/7 (14%) had no available result. Seroconversion was accompanied by rising ADA levels, and 3/4 (75%) achieved disease control. Those who seroconverted tended to have higher initial titres (range 28–195 AU/mL, median 75 AU/mL, IQR: 62–95) compared with those who remained antibody positive (12 and 41 AU/mL). The persistently AAA-positive patients had the lowest ADA levels among the cohort; however, both 2/2 (100%) achieved treatment success. One demonstrated a rise in ADA level, while the other individual experienced fall from 26.1 mg to 0.0 mg/L, paradoxically still achieving disease control. Both patients with missing post-escalation antibody data achieved disease control.

All 8/8 (100%) baseline AAA-negative patients remained negative, with post-escalation ADA levels available for 6/8 (75%) patients, ranging from 15.2–32.7 mg/L (median 20.4 mg/L, IQR 15.5–22.1) and 4/8 (50%) achieving disease control. Overall, escalation was associated with rising ADA drug levels and antibody seroconversion in more than half of the AAA-positive patients. However, treatment success occurred across all antibody and drug-level subgroups, and no consistent biomarker pattern reliably predicted outcome.

#### Visual acuity outcomes

**Fig. 2:**
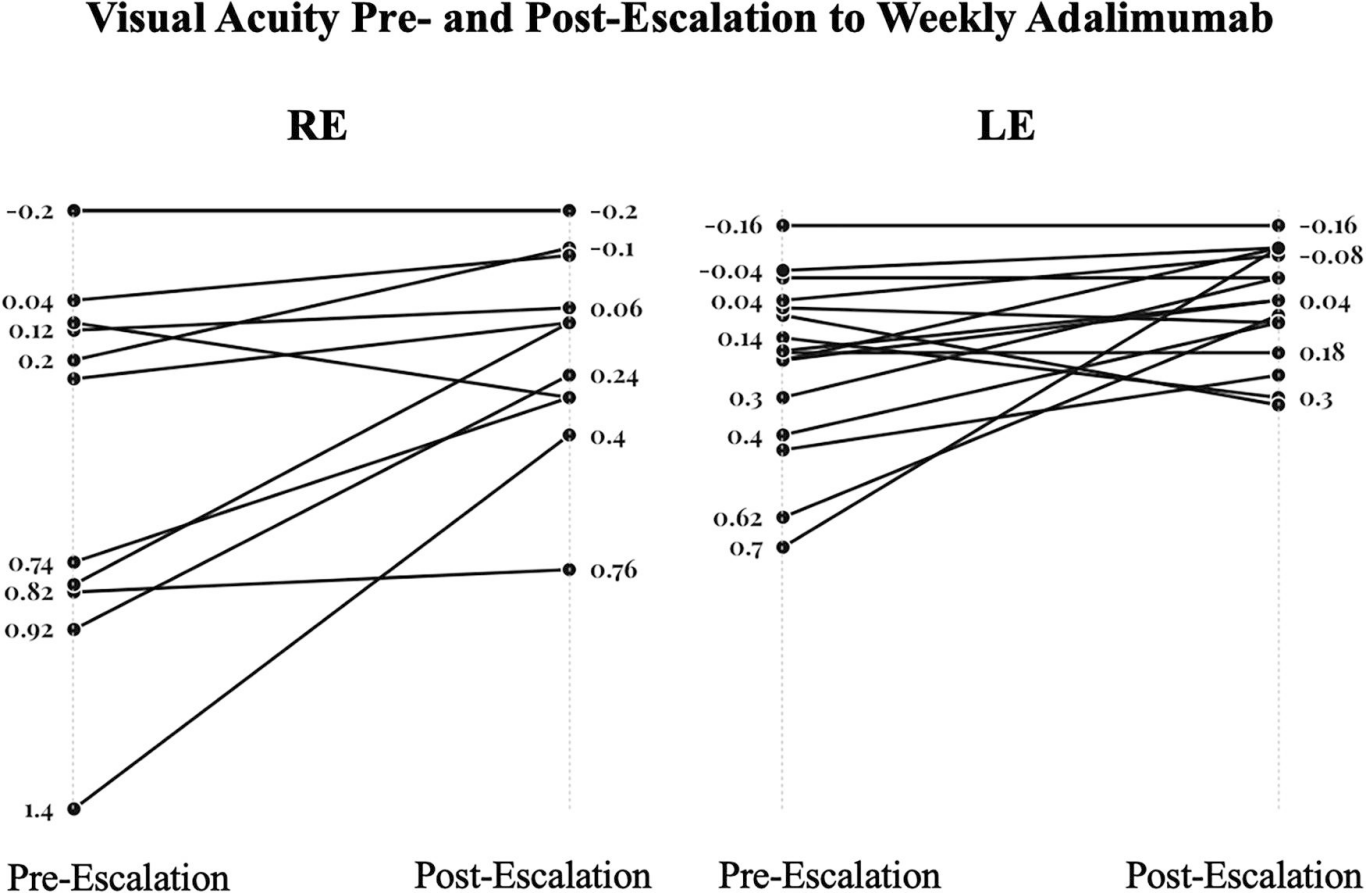
Visual acuity before and after escalation to weekly adalimumab (affected eyes only).

Visual acuity (VA) was recorded before and after escalation of ADA (Fig. 2). All 28 affected eyes from the 15 included patients were evaluable, with counting fingers (CF) and hand movements (HM) converted to estimated logMAR values [27]. Mean VA improved from 0.45 LogMAR (RE 0.56; LE 0.37) to 0.10 LogMAR (RE 0.15; LE 0.05) post-escalation. P8 underwent cataract surgery after starting weekly ADA, the first available VA following surgery was used for this analysis. Following escalation to weekly adalimumab, VA either improved or remained stable in the majority of affected eyes (26/28; 93%). 17/28 (61%) demonstrated improvement, 9/28 (32%) remained stable (defined as VA change <0.1logMAR), and 2/28 (7%) showed worsening of ≥0.1 logMAR. The mean change in visual acuity was an improvement of 0.36 logMAR units, with a median improvement of 0.14 logMAR. The largest improvement observed was 1.94 logMAR, while the largest decline was 0.2 logMAR. The patient with the most dramatic improvement was P8 who underwent cataract surgery and was amblyopic in her RE; this improvement was attributed to post-operative patching therapy rather than control of uveitis alone. P6 also exhibited a dramatic improvement of their VA explained by the resolution of their CMO.

When stratified by treatment response, affected eyes from patients who achieved clinical success (*n* = 19) demonstrated VA improvement in 12/19 (63%), with stable VA observed in 5/19 (26%) and 2/19 (11%) showing worsening. In the treatment failure group (*n* = 9) VA improved in 7/9 (78%) eyes and remained stable in 2/9 (22%), with no eyes demonstrating worsening. Although these proportions suggest variability in eye-level VA outcomes across both groups, the differences between groups were modest and did not indicate a clear separation in VA outcomes.

#### Topical and systemic steroid use

Prior to escalation to weekly adalimumab, all 15/15 (100%) patients required topical corticosteroid therapy for control of intraocular inflammation. Pre-escalation, the majority (12/15, 80%) required at least twice-daily dosing, including 5/15 (33%) receiving four or more drops per day, with several patients on more intensive regimens. At 3 months following escalation to weekly adalimumab, 11/15 (73%) patients required ongoing topical corticosteroids, while 4/15 (27%) discontinued steroid drops entirely. Of those who continued on topical steroids, 7/11 (64%) demonstrated a reduction in topical treatment frequency. 3/11 (27%) remained on unchanged dosing, and 1/11 (9%) required escalation of topical therapy during follow-up (P15) (Supplementary Table 1). No patients required systemic corticosteroids during the follow-up period.

#### Treatment tolerability

P12 discontinued weekly ADA after 9 months of treatment due to compliance difficulties and was subsequently switched to infliximab and MMF; however, this patient had achieved treatment success at 3 months. P15 discontinued adalimumab after one month in the context of poor adherence and repeated non-attendance; this patient was classified as treatment failure at 3 months. Excluding these two cases, all remaining patients were successfully taking weekly ADA at the latest review, with eight patients receiving concurrent MTX and six on MMF. These patients continued the same treatment regimens throughout follow-up period. No patient discontinued treatment due to adverse effects, and no treatment-related complications were reported.

## Discussion

This retrospective study demonstrates that escalation to weekly ADA enabled 66.7% of children with refractory pCAU to improve control of their eye inflammation. While the safety and efficacy of weekly ADA has been explored in the context of other inflammatory conditions including inflammatory bowel disease [19, 20] and dermatological disorders such as psoriasis [22, 28] and hidradenitis suppurativa [21, 23], evidence specific to uveitis has been largely limited to adult populations [13, 24]. Similarly, studies evaluating weekly ADA across rheumatological conditions, including JIA, remain limited [16, 25, 29]. To date, only three studies by Huard et al., Roberts et al. and Corell et al. have specifically evaluated weekly ADA in paediatric uveitis, evaluating cohorts of 11, 19 and 17 patients respectively. Our findings, in which 66.7% of patients achieved treatment success after escalation, are consistent with these reports and contribute further to this growing body of literature [1, 10, 16].

Huard et al. (2023) reported a 63.6% success rate at 3 and 6 months, with success defined by combined measures of inflammation reduction and sparing of corticosteroid drop use. Importantly, no serious systemic side effects were observed. Correll et al. (2019) specifically addressed the safety profile of weekly ADA in 60 children with various rheumatic diseases, including uveitis. They found that while infections were common (40% of patients), serious infections requiring hospitalisations were rare (5%), and autoimmune complications were uncommon (3%). Consistent with these findings, no serious infections or adverse events were observed in our cohort. One patient (P12) discontinued treatment due to injection intolerance and one patient (P15) discontinued due to poor compliance rather than adverse effects. Overall, these findings reinforce that weekly ADA is a safe and well-tolerated escalation strategy in the paediatric population.

Roberts et al. (2022) described similar rates of success, reporting 63% of children with refractory uveitis responding to weekly ADA after failing with standard biweekly dosing. Their study compared weekly ADA with infliximab and found both strategies effective, with similar time-to-control and recurrence rates. Notably, even patients who failed weekly ADA responded to infliximab, suggesting that escalation beyond weekly ADA with alternative biological treatments may be beneficial in some cases [30]. However, logistical considerations, such as intravenous access required for infliximab infusion, influenced patients and parents’ preference for ADA, highlighting its practical advantage in paediatric care. Additionally, studies from England, Spain and Canada have reported ADA to be a more affordable alternative to infliximab [31–33]. Although these comparisons were based on standard two-weekly dosing, they support the use of weekly ADA as a rational and accessible intermediate step before progressing to infusion-based therapies.

This study provides further evidence of the effectiveness and safety of weekly ADA and, in addition, explores the role of serum drug levels and AAA antibody status. AAA have been shown to be associated with lower serum drug concentrations and poorer clinical outcomes [34–36]. These studies used trough serum drug levels. ADA levels in this cohort were measured at random time points rather than trough levels, which may limit direct comparability with other studies. As expected, baseline AAA-positive patients demonstrated lower median ADA levels compared with AAA-negative patients (7.5 mg/L vs. 10.6 mg/L), with the two persistently seropositive patients having the lowest post-escalation ADA levels of the cohort. Higher antibody titres were generally associated with lower serum ADA levels (Spearman’s ρ = –0.71, *p* = 0.11). Nonetheless, treatment success was still achieved in 86% of baseline AAA-positive patients, indicating that control of inflammation is possible even in the presence of antibodies, possibly reflecting restoration of higher serum concentrations with dose escalation. Post-escalation, median ADA levels rose substantially compared to pre-escalation (18.1 mg/L vs. 10.1 mg/L); however, median drug levels were numerically higher in the treatment failure group compared to the success group (20.4 mg/L vs. 15.5 mg/L). There was no statistically significant association between pre-escalation (*p* = 0.89) or post-escalation (*p* = 0.32) ADA levels or AAA status (*p* = 0.28) and treatment success. One patient demonstrated a substantial fall in ADA concentration alongside persistent AAA positivity but nevertheless achieved treatment success by our definition, illustrating the variability in pharmacokinetic-clinical relationships. All baseline AAA-negative patients remained negative, with stable ADA levels and 50% achieving disease control.

Of the AAA-positive patients, 57% seroconverted to negative antibody status, accompanied by rising ADA levels, with 75% of these achieving disease control. This supports prior evidence that higher dosing may restore higher serum drug concentrations sufficient to suppress AAA production and subsequently reach therapeutic drug levels and overcome antibody-mediated loss of response [37]. We did not find a relationship between strength of antibody positivity and seroconversion or outcome, suggesting that even patients with substantial immunogenicity may respond to escalation.

Overall, neither antibody status nor serum ADA concentration alone reliably predicted clinical response in this cohort. Disease control was achieved in patients across subgroups, including 100% of the persistently antibody-positive patients and 50% of the seronegative patients. Similarly, although lower drug levels and higher antibody titre tended to cluster together, these were not consistent predictors of outcome. Notably, some non-responders exhibited high pre-escalation serum ADA concentrations, suggesting that lack of response may not be primarily pharmacokinetic in nature and that further dose escalation may therefore provide limited additional therapeutic benefit. These findings suggest that treatment is not determined by immunogenicity or serum concentration in isolation, but is likely influenced by additional factors such as concurrent systemic immunosuppression, genetic variability, and patient-specific characteristics [37]. One potential explanation is the heterogeneity of AAAs; not all detected antibodies are functionally neutralising, which may explain preserved drug activity and clinical response in some AAA-positive patients. Further large-scale, prospective studies are warranted to better define the role of AAAs and therapeutic drug monitoring in adalimumab therapy.

Visual acuity outcomes following escalation to weekly ADA were encouraging, with a cumulative 93% of affected eyes showing either improvement (17/28, 61%) or stability (9/28, 32%). VA gains occurred in both responders and non-responders, highlighting that visual function may improve even when formal criteria for treatment success are not fully met. While control of intraocular inflammation was the primary treatment goal, VA remains a key functional outcome, particularly in paediatric patients at risk of amblyopia and irreversible vision loss [38]. The positive visual outcomes observed in this cohort highlight the potential benefit of early escalation of treatment in refractory cases to minimise vision loss. Although gains in VA are multifactorial, they likely primarily reflect improved inflammatory control alongside resolution of uveitis-related complications such as macular oedema, supporting prompt escalation to prevent irreparable damage to vision [39].

Reducing topical corticosteroid dependence is a key therapeutic benefit of adalimumab, particularly given the well-established risk of steroid-related ocular complications such as treatment-resistant glaucoma and cataract formation [10]. In this study, we defined success as requiring fewer than two steroid drops per day, a quantity considered relatively safe. Following escalation, the majority of patients reduced their topical steroid burden (73%), with just over one quarter of patients discontinuing drops entirely by three months. Previous studies have demonstrated that patients using ≤2 drops daily do not have a significantly increased risk of developing cataracts, at similar level to normal population, although intraocular pressure elevation continues to be a concern on 2-3 drops per day, therefore <2/day remains the recommended threshold [10, 40, 41].

This study has limitations, including small sample size, retrospective design and variation in follow-up duration. An important limitation to note is the use of random serum adalimumab levels rather than true trough levels; samples obtained in closer proximity to drug administration may result in artificially elevated serum drug concentrations, potentially misrepresenting pharmacokinetic exposure and biasing assessment of the effect of escalation to weekly dosing. As such, post-escalation serum adalimumab levels should be interpreted with caution. This, together with the observational design, should be considered when interpreting the relationship between serum drug levels, antibody status and clinical outcomes.

Nonetheless, this work contributes to a growing body of evidence supporting weekly ADA as a safe, accessible and effective option for children with refractory uveitis. Larger prospective studies are warranted to clarify predictors of response and refine therapeutic drug monitoring strategies. However, despite the absence of definitive serological biomarkers on treatment outcomes, clinical escalation to weekly dosing offers a promising option for children with ongoing inflammation despite maximal standard care.

## Conclusion

In conclusion, weekly adalimumab dosing appears to be a safe, effective and well-tolerated option for children with pCAU refractory to standard dosing. Seroconversion from antibody-positive to negative status was observed in over half of affected patients and was mostly accompanied by rising drug levels and treatment success. Overall, neither antibody status nor drug levels consistently predicted outcome, with both treatment success and failure occurring across all subgroups. Biomarker interpretation remains complex and their utility in guiding treatment decisions is currently limited. This study adds to the growing body of evidence suggesting that weekly adalimumab may be effective in achieving disease control in persistent cases, even in the presence of immunogenicity. Further prospective studies are required to better understand biomarker correlations and confirm these findings in larger paediatric cohorts.

## Summary

### What was known before

- A subset of children with non-infectious uveitis remain refractory to standard biweekly adalimumab despite concurrent immunosuppression. Escalation to weekly adalimumab has shown benefit in small adult series, but paediatric data are limited. The role of serum adalimumab levels and anti-drug antibodies in predicting treatment response remains unclear.

### What this study adds

- Weekly adalimumab achieved disease control in two-thirds of children with refractory non-infectious uveitis across two UK tertiary centres. Treatment was safe and effective, including in patients with anti-adalimumab antibodies, with several antibody-positive patients seroconverting following escalation. Neither serum drug level nor antibody status consistently predicted response, indicating that clinical outcomes can improve despite immunogenicity.

## Supplementary information

Supplementary Table 1

Supplementary Material

## Data Availability

All data generated or analysed during this study are included in this published article [and its supplementary information files].
