# Supplementary material for: Clinical outcomes of weekly adalimumab in refractory non-infectious paediatric uveitis and the role of serum drug levels and anti-adalimumab antibodies
Source: Eye (Lond). 2026 May 29;40(11):1669–75. doi: 10.1038/s41433-026-04460-x (PMC13415550; doi:10.1038/s41433-026-04460-x)
Supplement: Supplementary file 2 — Supplementary Material [file 41433_2026_4460_MOESM2_ESM.docx]

**Supplementary Material**

- **Supplementary Table 1. Clinical characteristics of 15 paediatric patients escalated to weekly adalimumab.** Table includes age at escalation, laterality of uveitis, underlying diagnosis, concurrent systemic immunosuppression, use of topical corticosteroids, ocular complications, and best corrected visual acuity (VA) in right eye (RE) and left eye (LE) before and after escalation (logMAR). Abbreviations: TINU = tubulointerstitial nephritis and uveitis; JIA = juvenile idiopathic arthritis; NIU = non-infectious uveitis; MTX = methotrexate; MMF = mycophenolate mofetil; BE = both eyes; OD = once daily; BD = twice daily; AM = morning; PM = evening; PSC = posterior subcapsular cataract; ERM = epiretinal membrane; VH = vitreous haemorrhage; CMO = cystoid macular oedema; CNV = choroidal neovascularisation; RD = retinal detachment; CF = counting fingers; HM = hand movements.
